# Foodborne Pathogens: The Antimicrobial Resistance from Farm to Fork

**DOI:** 10.3390/pathogens14070632

**Published:** 2025-06-25

**Authors:** Gabriel Augusto Marques Rossi, Laryssa Freitas Ribeiro, Marita Vedovelli Cardozo

**Affiliations:** 1 Department of Veterinary Medicine, University Vila Velha (UVV), Av. Comissário José Dantas de Melo, n.21, Vila Velha 29102-920, ES, Brazil; 2 Mário Palmério University Center (UniFucamp), Av. Brasil Oeste, s/n, Jardim Zenith, Monte Carmelo 38500-000, MG, Brazil; laryssaribeiro84@gmail.com; 3 Department of Pathology, Reproduction and One Health, Faculty of Agricultural and Veterinary Sciences, São Paulo State University (UNESP), Jaboticabal 14884-900, SP, Brazil; marita.vedovelli@unesp.br

Foodborne diseases pose a major threat to global public health and contribute substantially to economic losses, increased healthcare expenses, negative effects on child development, reduced productivity, and losses in international trade [1]. The financial impact in the United States alone was estimated at around USD 75 billion in 2023 [2]. Each year, the consumption of unsafe food is linked to approximately 600 million cases of illness and 420,000 deaths worldwide, resulting in the loss of 33 million disability-adjusted life years (DALYs) [3]. These diseases are triggered by a variety of etiological agents, including bacteria, viruses, parasites, prions, and natural toxins. Despite this diversity, bacterial contamination remains the most common and impactful cause of foodborne diseases. While diseases caused by foodborne bacteria are sometimes mild and self-limiting, they can result in severe illness outcomes or death, particularly when treatment options are limited due to the growing challenge of antimicrobial resistance (AMR).

In 2021, AMR was associated with approximately 4.71 million deaths worldwide, including 1.14 million fatalities directly attributable to resistant bacterial infections. Forecasts suggest that by 2050, AMR could be the direct cause of nearly 1.91 million deaths and contribute to a total of 8.22 million deaths globally [4]. These alarming figures highlight the urgent need to adopt effective prevention and control strategies across the entire food production chain. Applying the “from farm to fork” concept is crucial to guarantee that food reaching consumers is safe and does not act as a vehicle of transmission for the spread of antimicrobial-resistant bacteria.

In this context, our Special Issue titled “Foodborne Pathogens: The Antimicrobial Resistance from Farm to Fork” aimed to compile manuscripts focused on evaluating bacterial AMR at different stages of various food production chains. The goals are to provide data that contribute to understanding the current scenario, to offer insights for future perspectives, and to support institutions responsible for addressing this serious threat to food safety and global public health.

Wang et al. (Contribution 1) investigated the connections between antimicrobial-resistant pathogens, resistance genes, and antimicrobial compounds linked to resistance in 6900 isolates obtained from chickens and turkeys in the United States. Their findings identified *Salmonella enterica* as the most frequently detected antimicrobial-resistant pathogen, while also emphasizing the relevance of other bacterial species such as *Campylobacter jejuni*, *Escherichia coli*, and *Shigella*. Additionally, the study revealed a high prevalence of resistance to tetracycline and streptomycin, alongside the frequent presence of resistance genes like *tet(A)*, *mdsA*, and *mdsB*. Spatial analysis conducted by these authors demonstrated the existence of localized clusters of resistant pathogens, resistance genes, and antimicrobial agents, suggesting clear geographic trends in the distribution of AMR.

Sampaio et al. (Contribution 2) focused on determining the prevalence and AMR profiles of 1260 *E. coli* isolates obtained from different stages of the swine production chain. This research also assessed the occurrence of extended-spectrum beta-lactamase (ESBL)-producing strains and compared resistance patterns among various sample types, including different slaughterhouse sites, feces, commercial pork cuts, environmental samples, and feces from workers. The analysis showed that 80.71% of the isolates were classified as multidrug-resistant (MDR). Furthermore, all isolates identified as ESBL producers (1.58%) displayed multidrug resistance, particularly to amoxicillin, tetracycline, and chloramphenicol. These results underscore the role of the swine production chain as a significant reservoir for MDR *E. coli* strains.

The third article is by Han et al. (Contribution 3), focusing on assessing AMR in *E. coli* isolates obtained from broiler carcasses, including strains classified as Extraintestinal Pathogenic *E. coli* (ExPEC), collected from wet markets and industrial processing facilities. The results demonstrated that 94.6% (54 out of 56) of the ExPEC isolates were MDR, showing resistance to more than three classes of antibiotics, a finding that poses serious concerns regarding the spread of AMR. Furthermore, the MALDI-TOF MS analysis revealed substantial genetic diversity among the ExPEC isolates, with no distinct clustering based on processing environment or sampling location. Considering these outcomes, the authors highlighted the necessity of promoting prudent antibiotic usage in poultry production to help mitigate the public health threats linked to ExPEC and AMR.

In the fourth article, written by Huang et al., a set of *Edwardsiella piscicida* isolates obtained from Largemouth Bass (*Micropterus salmoides*) in Guangdong, China, was evaluated for susceptibility to 11 antibiotics commonly employed in local aquaculture. The analysis revealed alarmingly high AMR rates, with 96.25% of the strains considered as resistant to ciprofloxacin, 60–63% to sulfonamides, 56.25% to thiamphenicol, 43.75% to florfenicol, 40% to enrofloxacin, 32.5% to doxycycline, 16.25% to flumequine, and 1.25% to neomycin. Moreover, 76.25% of the isolates exhibited resistance to more than two antibiotic classes. These outcomes underscore the urgent need for aquaculture workers to adopt best practices for antibiotic stewardship, including correct dosing, adequate treatment duration, and the rotation of antimicrobial agents. The study also highlights the critical role of reinforcing biosecurity protocols to limit the spread of resistant bacteria in aquaculture environments.

The fifth manuscript is a review authored by Viana et al. It is a comprehensive review of scientific studies that investigated the presence of *E. coli*, *Salmonella* spp., and *Staphylococcus* spp. isolated from food handlers, a critical yet often overlooked link in the food production chain concerning the dissemination of antimicrobial-resistant bacteria. The review provided insights into the persistence and transmission routes of these pathogens as well as the key challenges in their control. The AMR profiles reported in the included studies revealed worrisome patterns, including widespread resistance to β-lactam antibiotics and the emergence of resistance to critical drugs such as carbapenems. Of particular concern is the significant presence of MDR strains and ESBL-producing isolates, with an especially high prevalence in low- and middle-income countries. These authors identified several factors contributing to the spread of resistant bacteria among food handlers, such as insufficient hand hygiene practices, inadequate sanitation infrastructure, and the asymptomatic carriage of resistant strains. Based on these findings, the study reinforces the urgent need to implement robust surveillance programs, to develop effective decolonization strategies, to improve hygiene standards, and to strengthen training programs for food handlers to mitigate the transmission of antimicrobial-resistant pathogens along the food production chain.

Finally, the last contribution is by Delaporte et al. This literature review studied the aerotolerance mechanisms of *Campylobacter* spp., a microaerophilic pathogen that, despite its oxygen sensitivity, remains one of the leading causes of foodborne illness worldwide. These authors discussed how aerotolerance enables this microorganism to persist throughout the food production chain from farm to fork. They highlighted that aerotolerance is significantly influenced by various environmental conditions, such as the type of growth media, temperature, and interactions with other microorganisms. Moreover, biofilm formation, refrigeration, and low-light environments further contribute to a microorganism’s survival. The review also provides an overview of the genetic mechanisms currently associated with aerotolerance in *Campylobacter*, offering valuable insights into how this pathogen adapts and survives under stressful environmental conditions.

We believe that this Special Issue provides valuable contributions to the understanding of the current situation regarding the occurrence of antimicrobial resistant foodborne pathogens at various stages of the food production chains. Together, these studies can serve as a warning to health and food organizations, emphasizing the need to address this serious threat through a One Health approach that implements integrated preventive and control measures focused on human, animal, and environmental health.
